# Selection of waterlogging tolerant sesame genotypes (*Sesamum indicum* L.) from a dataset using the MGIDI index

**DOI:** 10.1016/j.dib.2024.110176

**Published:** 2024-02-08

**Authors:** Abul Fazal Mohammad Shamim Ahsan, Zakaria Alam, Faruque Ahmed, Sanjida Akter, Md. Anwar Hossain Khan

**Affiliations:** a Plant Physiology Division, Bangladesh Agricultural Research Institute, Gazipur 1701, Bangladesh; b Tuber Crops Research Centre, Bangladesh Agricultural Research Institute, Gazipur 1701, Bangladesh; c Entomology Division, Bangladesh Rice Research Institute (BRRI), Gazipur 1701, Bangladesh

**Keywords:** Waterlogging, Stress indices, Factor analysis, Broad sense heritability, Selection gain

## Abstract

The dataset explores the impact of waterlogging stress on sesame plants during the pre-flowering stage, recognizing its global impact on crop yield and the identification of tolerant genotypes using the MGIDI index. Carried out in Bangladesh, the research assesses the survival status, grain yield, and stress tolerance indices of 40 sesame genotypes, revealing that twelve of them demonstrated resilience under 72 h of waterlogging stress at the pre-flowering stage. There were variations in genotypic grain yield, and G15 exhibited the highest yields, recording 5.22 g/plant under normal conditions and 4.10 g/plant under waterlogging stress. The MGIDI index, evaluating waterlogging tolerance, identified G4 as the most favorable genotype, followed by G5 and G12. Factor analysis within the MGIDI index uncovered distinct tolerance and susceptibility indices, highlighting strengths and weaknesses in the selected genotypes. The selection gain percentages of these genotypes ranged from 12.9 to 37.4, indicating high broad-sense heritability (≥0.97). These results underscore the potential of genotype selection based on waterlogging stress indices, providing valuable insights for breeders addressing stress-related crop challenges in the face of changing climatic conditions.

Specifications Table

**Table:** 

Subject	Agricultural and Biological Science
Specific subject area	Agronomy and Crop Science
Data format	Raw
Type of data	Table and Figures
How the data were collected	The survival status of sesame genotypes was assessed following waterlogging stress at the pre-flowering stage. The grain yield data for plants subjected to stress and those not exposed to stress was obtained using an analytical balance. The total weight of grain (g) per genotype per replication was documented, and the results were averaged for analysis.
Data source location	The trial took place in the net house of RPRS, Bangladesh Agricultural Research Institute, Madaripur, Bangladesh, situated between 23°00′ and 23°30′ north latitudes and 89°56′ and 90°21′ east longitudes.
Data accessibility	Repository name: Mendeley Data Direct URL to data: https://data.mendeley.com/datasets/9sdfh8wmxc/1

## 1 Value of the Data

• The dataset provides valuable insights into how waterlogging stress affects sesame plants, particularly during the crucial pre-flowering stage. Grasping the worldwide consequences of this stress on crop yield is vital for formulating efficient crop management strategies applicable to both the sesame production sector and individual farmers.
• Utilizing the MGIDI index, the dataset imparts valuable details about genotypes that demonstrate tolerance in the face of waterlogging stress, supplying nuanced understandings into the strengths and weaknesses of selected genotypes. This information holds significant importance for breeders and researchers engaged in the selection and advancement of sesame varieties with improved tolerance to waterlogging.
• The dataset underscores pivotal elements in pinpointing waterlogging stress-tolerant sesame genotypes within a diverse germplasm collection. The chosen genotypes bring practical advantages, incorporating metrics like broad-sense heritability, selection differential, and selection gain, delivering a quantitative assessment of the data's reliability and prospective influence on forthcoming breeding initiatives.

## 2 Background

Sesame (*Sesamum indicum* L.), among the oldest and most vital oilseed crops globally [1], spans approximately 13 million hectares in cultivation, yielding around 6.5 million tons yearly [2]. Typically grown in rainfed conditions, sesame frequently encounters flooded environments [3]. Even short periods of excessive moisture significantly reduce grain yield [4]. Cultivating sesame on poorly drained soils can result in yield losses exceeding 40% within 36 days of emergence [5]. Crop yield reductions due to waterlogging, influenced by soil type and stress duration, can range from 15% to 80% [6]. Climate change may exacerbate these conditions, potentially increasing stress frequency and severity. Breeders face challenges in genotype selection amid these conditions. However, considering multiple traits, the concept of a plant ideotype may aid in identifying stress-tolerant genotypes within germplasm collections.

## 3 Data Description

The dataset presented in this article comprises one figure and three tables. Table 1 exhibits the survival status of forty sesame genotypes exposed to waterlogging stress and normal conditions. Notably, twelve genotypes demonstrated resilience during a 72-h stress period at the pre-flowering stage, while all forty genotypes survived in normal conditions.

**Table 1:** Survival status of forty sesame genotypes under normal and 72 hours waterlogging stress during pre-flowering stage.

Genotypes	Normal condition	72h stress	Genotypes	Normal condition	72 h stress
G1	√	√	G21	√	√
G2	√	×	G22	√	×
G3	√	×	G23	√	×
G4	√	√	G24	√	×
G5	√	√	G25	√	×
G6	√	×	G26	√	×
G7	√	×	G27	√	×
G8	√	×	G28	√	×
G9	√	×	G29	√	×
G10	√	×	G30	√	×
G11	√	×	G31	√	√
G12	√	√	G32	√	√
G13	√	×	G33	√	×
G14	√	√	G34	√	√
G15	√	√	G35	√	×
G16	√	√	G36	√	×
G17	√	×	G37	√	×
G18	√	×	G38^CK^	√	×
G19	√	√	G39^CK^	√	×
G20	√	×	G40^CK^	√	×

√survived; ×not survived

CK Check variety

Moving to Table 2, it presents the tolerance and susceptibility indices after 72 h of waterlogging, alongside the grain yield of 12 sesame genotypes. In normal conditions, genotype G15 exhibited the highest grain yield (5.22 g/plant), followed by G21 (5.20 g/plant) and G16 (5.03 g/plant). However, under stress conditions, G15 maintained the highest yield (4.10 g/plant), followed by G5 (3.89 g/plant) and G4 (3.73 g/plant). Table 3 depicts a factor analysis of selected genotypes, considering their 72-h waterlogged tolerance and susceptibility indices. Factor 1 (FA1) encompasses MP, GMP, STI, and YI, while Factor 2 (FA2) includes TOL, SSI, and YSI. The selection goal achieved a 100% success rate across all indices. The selected genotypes showed a selection gain percentage ranging from 12.9 to 37.5. Furthermore, the selection differential, based on predicted values, ranged from 0.12 to 0.58, carrying a high broad-sense heritability of ≥0.97.

**Table 2:** Calculated waterlogging stress tolerance and susceptibility indices along with grain yield of survived twelve sesame genotypes.

Genotypes	Y_p_^R^ (g/plant)	Y_s_^R^ (g/plant)	TOL^R^	MP^R^	GMP^R^	SSI^R^	STI^R^	YSI^R^	YI^R^
G1	2.80^12^	2.26^9^	0.54^2^	2.53^12^	2.52^12^	0.58^3^	0.32^12^	0.81^3^	0.76^9^
G12	4.57^7^	3.67^4^	0.90^4^	4.12^6^	4.10^5^	0.59^4^	0.84^5^	0.80^4^	1.23^4^
G14	4.73^6^	3.61^5^	1.12^6^	4.17^3^	4.13^3^	0.71^7^	0.86^3^	0.76^7^	1.21^5^
G15	5.22^1^	4.10^1^	1.12^7^	4.66^1^	4.63^1^	0.64^6^	1.08^1^	0.79^6^	1.38^1^
G16	5.03^3^	2.21^11^	2.82^12^	3.62^7^	3.33^8^	1.68^11^	0.56^8^	0.44^11^	0.74^11^
G19	4.40^9^	2.57^8^	1.83^8^	3.48^9^	3.35^7^	1.24^9^	0.56^7^	0.59^9^	0.86^8^
G21	5.20^2^	3.13^6^	2.07^9^	4.17^4^	4.03^6^	1.19^8^	0.82^6^	0.60^8^	1.05^6^
G31	4.31^10^	1.50^12^	2.81^11^	2.91^10^	2.54^11^	1.95^12^	0.32^11^	0.35^12^	0.50^12^
G32	4.93^4^	2.23^10^	2.70^10^	3.58^8^	3.31^9^	1.64^10^	0.55^9^	0.45^10^	0.75^10^
G34	2.90^11^	2.80^7^	0.10^1^	2.85^11^	2.85^10^	0.11^1^	0.41^10^	0.96^1^	0.94^7^
G4	4.52^8^	3.73^3^	0.79^3^	4.13^5^	4.11^4^	0.52^2^	0.85^4^	0.83^2^	1.25^3^
G5	4.93^4^	3.89^2^	1.04^5^	4.41^2^	4.38^2^	0.63^5^	0.96^2^	0.79^5^	1.31^2^

R Rank of genotypes; $Y_{S}$ genotype mean grain yield under stress condition; $Y_{P}$ genotype mean grain yield under non-stress condition; ${\bar{Y}}_{S}$ all genotypes’ mean grain yield under stress condition; ${\bar{Y}}_{P}$ all genotypes’ mean grain yield under non-stress conditions; TOL tolerance index; MP Mean Productivity; GMP Geometric Mean Productivity; SSI Stress Susceptibility Index; STI Stress Tolerance Index; YI Yield Index and YSI Yield Stability Index.

**Table 3:** Factor contribution, broad-sense heritability (h^2^), selection differential (SD) and selection gain (SG) obtained using MGIDI selection index of three waterlogging tolerant sesame genotypes.

Index	Factor	$\overset{‾}{X}$_o_	$\overset{‾}{X}$_s_	SD	h^2^	SG	Sense	Goal
MP	FA1	3.72	4.21	0.49	0.98	12.9	increase	100
GMP	FA1	3.61	4.18	0.58	0.98	15.7	increase	100
STI	FA1	0.68	0.88	0.20	0.98	29.5	increase	100
YI	FA1	1	1.26	0.26	0.98	25.4	increase	100
TOL	FA2	1.49	0.93	-0.56	0.97	-36.3	decrease	100
SSI	FA2	0.96	0.59	-0.37	0.98	-37.4	decrease	100
YSI	FA2	0.68	0.80	0.12	0.98	17.5	increase	100

$\bar{X}$_o_= observed mean, $\bar{X}$_s_=predicted mean

**Fig. 1:**
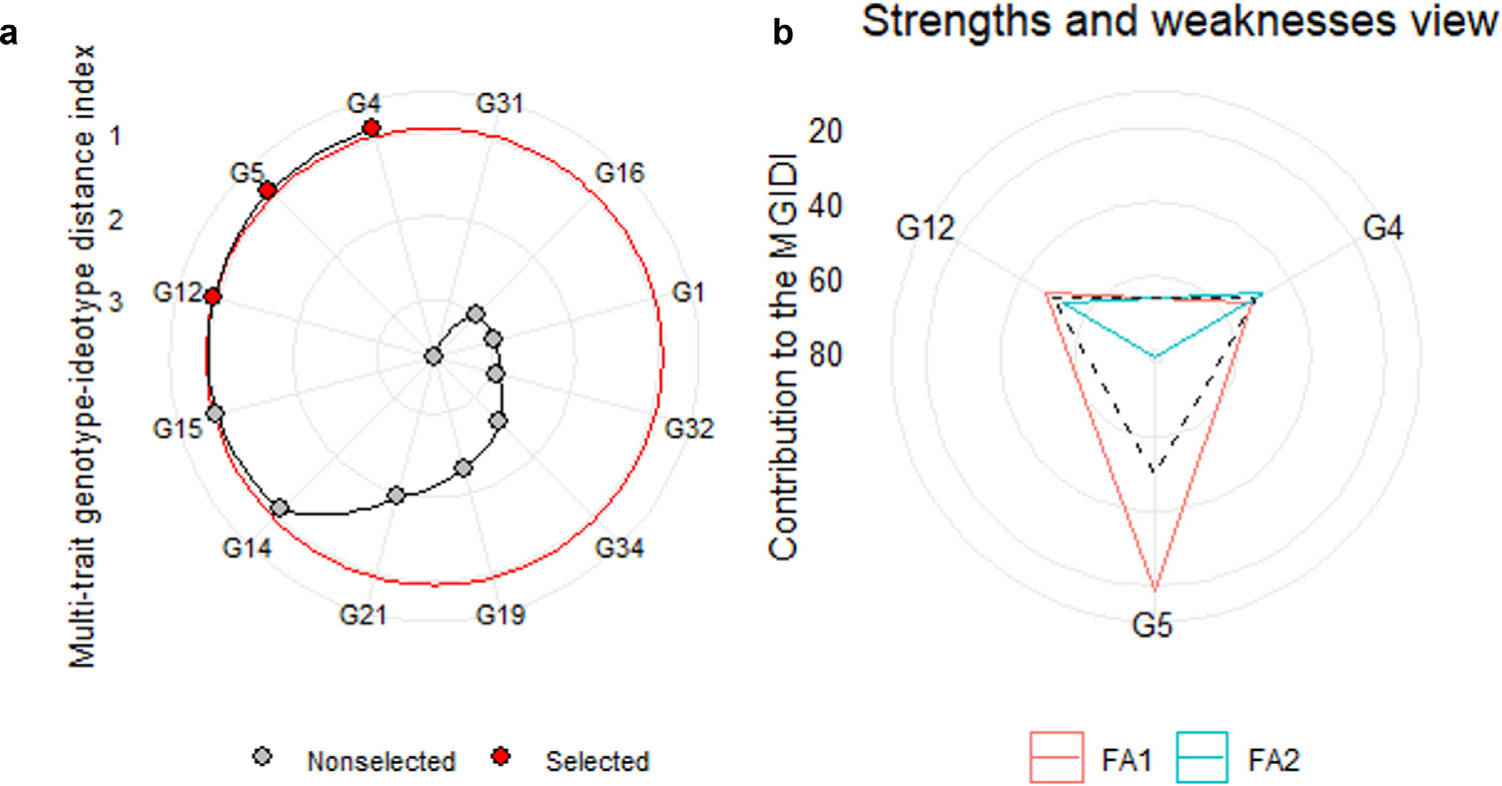
Selection of waterlog tolerant sesame genotypes through MGIDI index (a) and the strength and weaknesses of selected genotypes (b).

Fig. 1 depicts the ranking of 12 sesame genotypes based on waterlogging stress tolerance and susceptibility indices using the MGIDI index. In Fig. 1a, these genotypes are arranged in descending order of MGIDI index values, with the highest value at the centre and the lowest at the outer circle. The red circle represents the selection threshold (SI = 25%) set by the MGIDI selection index. Genotypes were selected based on their MGIDI index, as indicated by red dots. G4 emerged as the most desirable genotype, followed by G5 and G12, according to the MGIDI index. Fig. 1b illustrates the strengths and weaknesses of these selected genotypes. The positioning of factors relative to genotypes indicates their influence, while dotted lines represent average performance in factor contribution. Higher factor values moving toward the center indicate weaknesses, while lower values signify strengths. For instance, G5 and G12 exhibited notable strengths, contributing above average to FA1, unlike G4, which showed below-average contribution to FA1, emphasizing its weaknesses. Conversely, G4 demonstrated strengths by contributing above average to FA2, unlike G5 and G12, which exhibited below-average contribution, highlighting their weaknesses.

## 4 Experimental Design, Materials and Methods

### 4.1 Experimental treatments, design and plant materials

The study was conducted using a Randomized Complete Block design, comprising three replications. Each replication included two sets of 40 genotypes, comprising 37 accessions and 3 check varieties (Supplementary Table 1): one set under normal conditions and the other subjected to waterlogging stress, totalling 240 plastic plots. In one set containing 120 pots with 40 genotypes, waterlogging was induced 40 days after sowing (pre-flowering stage) by submerging them in a cement concrete water tub. The waterlogged conditions were maintained for 72 h, with the water level kept approximately 5 cm above the soil surface of the pots. After the waterlogging treatment, the water was drained, and plants were allowed to grow under normal conditions until maturity. Another set of 40 sesame genotypes was kept under normal conditions and maintained with standard management practices throughout the growing period until maturity. Harvesting for both sets was conducted at 96 days after sowing.

### 4.2 Experimental techniques

On March 13, 2018, plastic pots with dimensions of a top diameter of 20 cm, bottom diameter of 15 cm, and height of 19 cm were filled with a well-mixed combination of sandy loam soil and cow dung in a 4:1 ratio (6 kg per pot). A total of 2400 seeds belonging to 40 sesame genotypes were then sown in 240 pots, with each pot containing ten seeds. Fertilizers (100 kg/ha of N, 30 kg/ha of P, 55 kg/ha of K, 25 kg/ha of S, 3 kg/ha of Zn, and 1 kg/ha of B) were applied at sowing. After twenty days from sowing, excess seedlings were thinned, and three plants per pot were retained to grow.

### 4.3 Data collection and data analysis

Observations on the survival status of 40 sesame genotypes were recorded 20 days after draining water from pots. Simultaneously, the survival status of plants under controlled conditions was also assessed. Grain yield (g) for each genotype under both control (Yp) and stressed (Ys) conditions was measured per replication using an analytical balance and then averaged. Seven indices evaluating stress tolerance and susceptibility, including Tolerance Index (TOL), Mean Productivity (MP), Geometric Mean Productivity (GMP), Stress Susceptibility Index (SSI), Stress Tolerance Index (STI), and Yield Index (YI) (as shown in Table 4), were calculated using Microsoft Excel. Corresponding rankings for these indices were also determined. These computations were solely derived from the count of genotypes that survived under stress condition. To identify waterlogging stress-tolerant genotypes using the MGIDI index (Table 5), ideotype design and rescaling (Eq. viii) of stress tolerance and susceptibility indices were performed. For ideotype designing, the selection sense was increased for all indices except TOL and SSI in the MGIDI index (Eq. ix). Subsequently, variance components obtained from MGIDI analysis were utilized to calculate broad-sense heritability ($h^{2}$) (Eq. x) based on genotype means. The mean values and predicted BLUP (Best Linear Unbiased Prediction) values of seven indices (Supplementary Table 2) were computed to determine the selection differential (SD) (Eq. xi) and selection gain (SG) (Eq. xii). Factor analysis (xiii) was conducted to group traits (indices) and calculate factor loadings (Eq. xiv), which were then utilized to identify strengths and weaknesses (Eq. xv) in the selected genotypes. The ‘metan’ package in the R software was used to analyze and select the best-performing genotypes for waterlogging tolerance [7].

**Table 4:** Mathematical formulas of seven waterlogging stress tolerance and susceptibility indices.

Index	Equations	Equation No.	Reference
Tolerance Index (TOL)	$\text{TOL}=Y_{P}-Y_{S}$	i	[8]
Mean Productivity (MP)	$MP=\frac{Y_{p}+Y_{s}}{2}$	ii	
Geometric Mean Productivity (GMP)	$\text{GMP}=\sqrt{Y_{S}\times Y_{P}}$	iii	[9]
Stress Susceptibility Index (SSI)	$\text{SSI}=\frac{1-(Y_{S}/Y_{P})}{1-({\bar{Y}}_{S}/{\bar{Y}}_{P})}$	iv	[10]
Stress Tolerance Index (STI)	$STI=\frac{Y_{S}\times Y_{P}}{{\left(Y_{p}\right)}^{2}}$	v	[9]
Yield Index (YI)	$\text{YI}=\frac{Y_{s}}{{\bar{Y}}_{s}}$	vi	[11]
Yield Stability Index (YSI)	$YSI=\frac{Y_{S}}{Y_{P}}$	vii	[12]

**Table 5:** The equations to select best performing waterlogging stress tolerant sesame genotypes including their strengths and weaknesses, broad sense heritability (*h*^2^), selection gain (SG) and factor analysis.

Index	Equations	Equation No.	Reference
Ideotype design and rescaling of traits	$rX_{ij}=\frac{\eta_{nj}-\varphi_{nj}}{\eta_{oj}-\varphi_{oj}}\times \left(\theta_{ij}-\eta_{0j}\right)+\eta_{nj}$	viii	[13]
Multi trait genotype-ideotype index (MGIDI)	$MGIDI_{i}={\left[\sum_{j=1}^{f}{\left(\gamma_{ij}-\gamma_{j}\right)}^{2}\right]}^{0.5}$	ix	
Broad sense heritability (*h*^2^)	$h^{2}={\hat{\sigma }}_{a}^{2}/\left({\hat{\sigma }}_{a}^{2}+{\hat{\sigma }}_{\varepsilon }^{2}/r\right)$	x	
Selection differential (SD)	$SD={\overset{‾}{X}}_{s}-{\overset{‾}{X}}_{o}$	xi	
Selection gain (SG)	$SG\left(\%\right)=\frac{\left({\overset{‾}{X}}_{s}-{\overset{‾}{X}}_{o}\right)\times h^{2}}{{\overset{‾}{X}}_{o}}\times 100$	xii	
Factor analysis	X=μ+Lf+ε	xiii	
Factor loadings	$F=Z{\left(A^{T}R^{-1}\right)}^{T}$	xvi	
Strength and weaknesses of selected genotypes	$\omega_{ij}=\frac{\sqrt{D_{ij}^{2}}}{\sum_{j=1}^{f}\sqrt{D_{ij}^{2}}}$	xv	

## Limitations

A drawback in this dataset is the potential lack of genuine replication of real-world field conditions. While the controlled environment provides precise measurements, the inherent variability in actual field conditions, affected by elements such as soil types, microclimates, and agronomic practices, presents a challenge. As a result, the ability to apply the study's findings to practical agricultural settings may be limited. To improve the practical usefulness of the identified tolerant genotypes, it is crucial to conduct additional validation across a variety of diverse field conditions.

## Ethics Statement

All authors have read and follow the ethical requirements for publication in Data in Brief and our work meets these requirements. Our work does not involve studies with animals and humans.

## CRediT authorship contribution statement

**Abul Fazal Mohammad Shamim Ahsan:** Conceptualization, Methodology, Investigation, Supervision. **Zakaria Alam:** Software, Visualization, Writing – original draft, Writing – review & editing. **Faruque Ahmed:** Investigation, Supervision, Validation, Software. **Sanjida Akter:** Data curation, Software. **Md. Anwar Hossain Khan:** Methodology, Data curation.

## Data Availability

Selection of pre-flowering waterlogging stress-tolerant genotypes from a dataset of sesame (Sesamum indicum L.) using the MGIDI index (Original data) (Mendeley Data)

## Appendix Supplementary materials

Image, application 1
