# Supplementary material for: Selection of waterlogging tolerant sesame genotypes (Sesamum indicum L.) from a dataset using the MGIDI index
Source: Data Brief. 2024 Feb 8;53:110176. doi: 10.1016/j.dib.2024.110176 (PMC10875224; doi:10.1016/j.dib.2024.110176)
Supplement: Supplementary file 1 [file mmc1.docx]

Supplementary Table 1. List of forty sesame genotypes with accession number

| **Genotype code** | **Accession Number** | **Genotype code** | **Accession Number** |
| --- | --- | --- | --- |
| G1 | BD 6997 | G21 | BD 6996 |
| G2 | JP 01811 | G22 | BD 6999 |
| G3 | GP 35-1 | G23 | BD 6968 |
| G4 | Ses-MR-20 | G24 | JP 01711 |
| G5 | BD 6998 | G25 | JP 14004 |
| G6 | BD- 6971 | G26 | BD 6984 |
| G7 | BD 6966 | G27 | BD 6989 |
| G8 | BD 7008 | G28 | JP 01411 |
| G9 | BD 7021 | G29 | BD 6978 |
| G10 | BD 6959 | G30 | BD 7004 |
| G11 | JP 00411 | G31 | BD 6991 |
| G12 | BD 7012 | G32 | BD 6995 |
| G13 | BD 6988 | G33 | BD 6994 |
| G14 | JP 01611-2 | G34 | BD 7000 |
| G15 | BD 6985 | G35 | BD 7005 |
| G16 | BD 6981 | G36 | GP 01311 |
| G17 | GP 14003 | G37 | BD-10165 |
| G18 | GP 5 | G38 | BARI Til 3 |
| G19 | JP 01514 | G39 | BARI Til 4 |
| G20 | BD 10167 | G40 | BINA Til 3 |

Supplementary Table 2. Calculated BLUP values of waterlogging stress tolerance and susceptibility indices for twelve sesame genotypes

| **Genotypes** | **TOL** | **MP** | **GMP** | **SSI** | **STI** | **YSI** | **YI** |
| --- | --- | --- | --- | --- | --- | --- | --- |
| **G1** | 0.57 | 2.55 | 2.54 | 0.59 | 0.33 | 0.80 | 0.76 |
| **G12** | 0.92 | 4.11 | 4.09 | 0.60 | 0.84 | 0.80 | 1.23 |
| **G14** | 1.13 | 4.16 | 4.12 | 0.71 | 0.85 | 0.76 | 1.21 |
| **G15** | 1.13 | 4.64 | 4.61 | 0.65 | 1.07 | 0.78 | 1.37 |
| **G16** | 2.78 | 3.62 | 3.34 | 1.66 | 0.56 | 0.45 | 0.75 |
| **G19** | 1.82 | 3.49 | 3.36 | 1.23 | 0.57 | 0.59 | 0.87 |
| **G21** | 2.05 | 4.16 | 4.03 | 1.19 | 0.82 | 0.60 | 1.05 |
| **G31** | 2.77 | 2.92 | 2.56 | 1.93 | 0.33 | 0.36 | 0.51 |
| **G32** | 2.66 | 3.58 | 3.32 | 1.62 | 0.55 | 0.46 | 0.76 |
| **G34** | 0.15 | 2.87 | 2.86 | 0.13 | 0.42 | 0.96 | 0.94 |
| **G4** | 0.81 | 4.12 | 4.10 | 0.53 | 0.84 | 0.82 | 1.25 |
| **G5** | 1.06 | 4.40 | 4.36 | 0.64 | 0.96 | 0.79 | 1.30 |
| **Significance** | * | | | | | | |

*Genotype effect are significant at *p* < 0.05 for all variables in Likelihood Ratio Test
